# Efficacy of thermotherapy for herpes zoster and postherpetic neuralgia: A protocol for systemic review and meta-analysis

**DOI:** 10.1097/MD.0000000000023823

**Published:** 2021-01-08

**Authors:** Zhuang Li, Yalin She, Zhenke Luo, Zijun Liu, Wenya Pei, Jingchun Zeng, Guohuo Lin

**Affiliations:** a Guangzhou University of Chinese Medicine, Guangzhou; b The First Affiliated Hospital of Shenzhen University, Shenzhen; c Department of Acupuncture, The First Affiliated Hospital of Guangzhou University of Chinese Medicine; d Department of Acupuncture, The First Affiliated Hospital, Sun Yat-sen University, Guangzhou, Guangdong, China.

**Keywords:** fire needle, herpes zoster, moxibustion, postherpetic neuralgia, protocol, systematic review, thermotherapy

## Abstract

**Background:**

Herpes zoster (HZ), is a painful skin rash disease with cutaneous symptoms and acute zoster-associated pain (ZAP). Postherpetic neuralgia (PHN), as the most frequent sequela of HZ, can persist a long time. Both HZ and PHN may significantly impact the quality of life and made great economical afford to affected patients. Its optimal treatment on HZ and PHN is still an urgent problem. In China, thermotherapy, including moxibustion and fire needle, is widely used because they can quickly promote the recovery of shingles and reduce the occurrence of PHN. Thermotherapy can also reduce pain intensity, relieve anxiety, and improve quality of life of PHN. Based on the current literatures, the effect and safety of thermotherapy will be systematically evaluated to provide appropriate complementary therapies for HZ and PHN.

**Methods:**

Studies search for eligible randomized controlled trials (RCTs) that use thermotherapy including fire needle and moxibustion for HZ or PHN from the following databases: PubMed, EMBASE, Web of Science, the Cochrane Library, China National Knowledge Infrastructure (CNKI), China Biology Medicine Database (CBM), Technology Periodical database (VIP), and Wanfang database. Language restrictions for retrieving literature are English and Chinese. Their data extraction will be done by 2 researchers. Mean difference (MD) or relative risk (RR) with fixed or random effect model in terms of 95% confidence interval (CI) will be adopted for the data synthesis. To evaluate the risk of bias, the Cochrane's risk of bias assessment tool will be utilized. The sensitivity or subgroup analysis will also be conducted when meeting high heterogeneity (*I*^2^ > 50%).

**Results:**

This meta-analysis will provide an authentic synthesis of the thermotherapy's effect on HZ and PHN, including incidence of postherpetic neuralgia and adverse events.

**Discussion:**

The findings of the review offer updated evidence and identify whether thermotherapy can be an effective treatment for HZ and PHN for clinicians.

**Registration number:**

INPLASY2020110009.

## 1 Introduction

Herpes zoster (HZ), also named Shingles, is an infectious disease caused by reactivation of varicella-zoster virus (VZV). Cutaneous symptoms and acute zoster-associated pain (ZAP) affects quality of life (QoL) of the affected patients.

Postherpetic neuralgia (PHN) is refractory neuralgia that can persist for a long time and has severe pain left after the onset of HZ. A systematic review concealed that the incidence rate of HZ ranged between 3 and 5/1000 person-years in North America, Europe, and Asia-Pacific.^[1]^ It is found that the pain and discomfort associated with HZ and PHN can have a significantly negative and widespread impact on patients’ health related quality of life (HRQoL) and patients’ ability to engage in activities of daily living.^[2–5]^ Therefore, HZ and PHN made great economic burden. The healthcare costs and loss of production costs were between 100 and 175 euros per 3 months for the significant pain group after HZ in Netherlands.^[2]^ Some patients with PHN last for more than 10 years,^[6]^ affecting sleep and causing depression. Exclude immunocompromised patients, 6.6% of patients ≥50 years with HZ developed PHN in USA. PHN patients range from 50s to 80s spend from $6133 to $11,147 every year, which increases with age.^[7]^ 30% to 50% of patients with PHN experienced pain lasting for more than 1 year.^[1]^ It is necessary to find effective, low-toxic, and easy to operate treatments.

However, there was no obvious effective Western medicine treatment on HZ and PHN and Western medicine has certain side effects.^[8–10]^ The use of antiviral medications and glucocorticoids does not reduce the incidence of PHN.^[11]^ Thermotherapy, including fire acupuncture and moxibustion, have been used in clinical practice for more than 2000 years, recorded in Huangdi Nei Jing. Records of herpes zoster symptoms in ancient Chinese books appeared in the Pre-qin Dynasty's “Fifty-two prescriptions”^[12]^ earlier than Huangdi Nei Jing. Acupuncture was recommended as the treatment method for HZ and PHN in the 2018 Chinese Consensus of Herpes Zoster and the 2016 Chinese Consensus of Diagnosis and Treatment of Postherpetic Neuralgia.^[13,14]^ Acupuncture may reduce pain intensity, relieve anxiety, and improve quality of life in patients with PHN.^[15]^ Several researches proved fire acupuncture or moxibustion can significantly relieve pain, shorten the pain duration, and reduce the incidence of postherpetic neuralgia.^[16,17]^

However, there are few meta-analysis on the effect of fire acupuncture and moxibustion on HZ or PHN. The purpose of this meta-analysis is to assess the quality of current randomized controlled trials and further systematically evaluate the effectiveness and safety of fire acupuncture and moxibustion. This research may provide useful information in forming an optimal fire acupuncture or moxibustion treatment protocol.

## 2 Methods

The protocol for this systematic review was registered on INPLASY (INPLASY2020110009) and is available in full on the inplasy.com (https://doi.org/10.37766/inplasy2020.11.0009). The protocol follows the Preferred Reporting Items for Systematic Reviews and Meta-Analyses Protocols (PRISMA-P) statement guidelines. We will describe the changes in the full review if necessary.

### 2.1 Inclusion criteria for study selection

#### 2.1.1 Types of studies

All the randomized controlled trials (RCTs) of thermotherapy including fire needle and moxibustion as the treatment in English and Chinese for Herpes zoster or PHN will be involved in the review.

#### 2.1.2 Types of participants

Patients included will be clinically diagnosed with herpes zoster or PHN and there is no restriction on sex, age, race, or duration of the disease. Besides, studies that do not mention diagnostic criteria and with other specific primary diseases will be excluded.

#### 2.1.3 Types of interventions

One of thermotherapy, fire acupuncture, and moxibustion must be applied in the treatment group, while Western medicine must be applied in the control group with all types of point combination being acceptable.

#### 2.1.4 Types of outcome measures

##### 2.1.4.1 Primary outcomes

1. Total effective rate;
2. Pain intensity (measured by Visual Analogue Scale or other scales);
3. Recovery span of skin damage;
4. The duration of the pain.

##### 2.1.4.2 Secondary outcomes

1. Incidence of postherpetic neuralgia;
2. Adverse events.

### 2.2 Search methods for the identification of studies

#### 2.2.1 Electronic searches

The data collection will be conducted systematically by 2 researchers through 8 databases from their inception to Feb 5, 2020. The databases to be searched for eligible RCTs are as follows: PubMed, EMBASE, Web of Science, the Cochrane Library, China National Knowledge Infrastructure (CNKI), China Biology Medicine Database (CBM), Technology Periodical database (VIP), and Wanfang database. A combination of keywords, written in English and Chinese, will be searched, including: “thermotherapy,” “fire needle,” “moxibustion,” “herpes zoster,” and “postherpetic neuralgia.”

#### 2.2.2 Other searches

Despite published studies in journals, literatures from conference papers and references, will be searched.

### 2.3 Data collection and analysis

#### 2.3.1 Selection of studies

**Figure 1:**
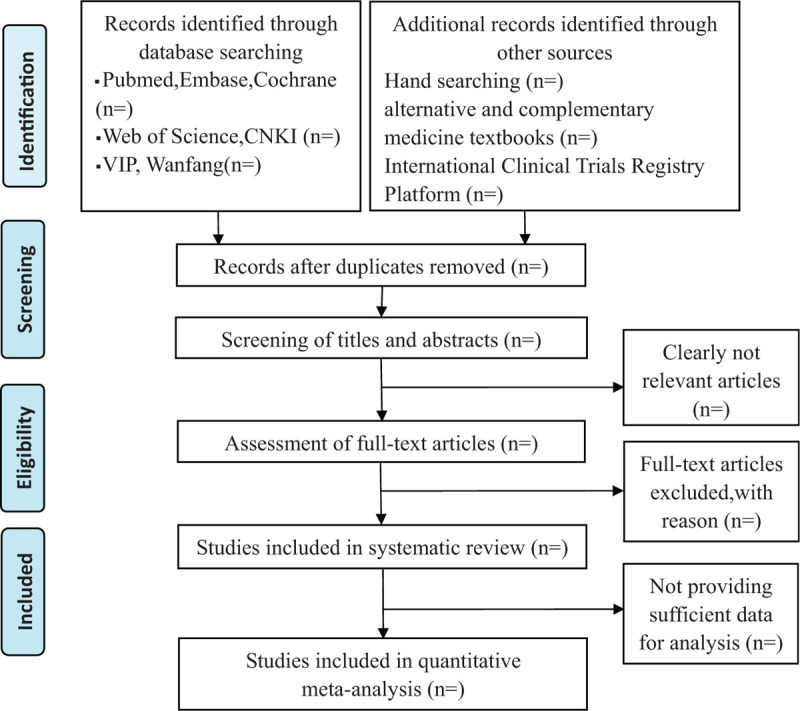
The PRISMA flow chart.

The inclusion and exclusion of studies will be accomplished by 2 reviewers (ZkL and ZL) independently and be crosschecked. First, reviewers will review the titles and abstracts of the literatures and exclude the markedly ineligible ones. Second, the full text will be carefully inspected for inclusion in the RCT trials through inclusion and exclusion studies. Any disagreement will be resolved through discussion with the third researcher. The whole selection process will be presented in a PRISMA flow diagram (Fig. 1).

#### 2.3.2 Data extraction and management

Study characteristics, details of the intervention and comparator used, outcome measures, results, and risk of bias ratings will be extracted by 2 individuals working independently, stored in Excel files and analyzed using RevMan 5.3 and Stata 13.0. Inconsistencies will be resolved through discussion and consultation with a third researcher.

#### 2.3.3 Dealing with the problematic data

When meeting problematic data which is ambiguous, contradictory, incorrect, or missing, the researcher will contact the first author for the clarified, correct, or omission data. The potential impact of suspicious data will be discussed additionally in Section 3.

#### 2.3.4 Assessment of risk of bias in included studies

Two reviewers will independently evaluate the methodological quality of included studies using the Cochrane Handbook's Risk of Bias Tool. Seven sectors will be evaluated, including random sequence generation, allocation concealment, blind participants and personnel, blind evaluation of results, completeness of result data, selective reporting, and other sources of bias. The reviewers will use 3 letters to indicate the results of the 3 levels of evaluation: “L” (low-risk), “U” (uncertain), “H” (high-risk). When there is different idea between 2 reviewers, a discussion with a third reviewer will be needed and the corresponding author will be connected when needed.

#### 2.3.5 Measures of treatment effect

Evaluating the effect of continuous data, the mean difference (MD) expressed as a 95% confidence interval (CI) will be used, just like the relative risk (RR) of dichotomous data.

#### 2.3.6 Assessment of heterogeneity

According to the data heterogeneity analysis, given by the *I*^2^ statistics, a random effect model or a fixed effect model is adopted. Specifically, if the heterogeneity is high (*I*^2^ > 50%), a fixed effect model will be used. Otherwise, a random effects model will be applied.

#### 2.3.7 Assessment of reporting bias

When the study is sufficient (at least 10 RCTs), the funnel graph will visually show the reported bias. If it shows asymmetry, then the test of Begg and Egger will be completed, and the value of *P* > .05 will be interpreted as no substantial reporting bias. Since the asymmetry of the funnel chart cannot replace publication bias, the potential reasons will be distinguished by small sample size, low methodological quality, or true heterogeneity.

#### 2.3.8 Data synthesis

The systematic review will be conducted with the use of RevMan 5.3. Taking account of the heterogeneity assessment, MD or RR with fixed or random effect model will be computed. Additionally, if heterogeneity is considered significant, the sensitivity or subgroup analysis will be generated to distinguish the source of it. When it comes to the situation that the data are insufficient for quantitative analysis, the review will only represent and summarize the evidence.

#### 2.3.9 Sensitivity analysis

As mentioned above, sensitivity analysis will be performed when the heterogeneity is greater than 50%. Specifically, despite studies of methodological quality, sample size, or older age, effect of missing data, a meta-analysis will be conducted again to determine whether these factors will affect the results.

#### 2.3.10 Subgroup analysis

After observing the obvious heterogeneity (*I*^2^ > 50%), a subgroup analysis will also be performed. Participant characteristics such as age and gender, pain degree, study quality, ethnicity, outcome measures, and other characteristics will become the content of the subgroup analysis.

#### 2.3.11 Quality of evidence

To assess the quality of evidence more objectively, reviewers will use the “Recommendation Assessment, Development, and Assessment Level (GRADE)” and fill out the “Summary of Survey Results” form.

#### 2.3.12 Ethics and dissemination

Since this protocol is for a systematic review, which involves no privacy data, ethical approval, and informed consent are needless. The results of the review will be widely disseminated through publications and conference presentations submitted to peer review.

## 3 Discussion

Shingles (herpes zoster) is an infectious skin disease caused by the reactivation of varicella-zoster virus (VZV), which is latent in the posterior root ganglion or cranial ganglion of the spinal cord. Researches proved that the degree of pain is closely related to mood disturbance (*r* = 0.846), sleep (*r* = 0.774), and QoL.^[3,18]^

The treatment goal of herpes zoster is to relieve pain in the acute phase, shorten the duration of the lesion, prevent the lesion from spreading, and prevent or reduce PHN and other complications. The purpose of PHN treatment is to effectively control pain as early as possible, relieve accompanying sleep and affective disorders, and improve the quality of life. However, their efficacy is still limited by Western medicine and have several adverse events^[5–7,19]^ and there is remaining considerable confusion about the optimal treatment for HZ and PHN.

In recent years, clinical practices and some studies have also found that thermotherapy, including fire acupuncture and moxibustion could offer great benefits to HZ and PHN and thermotherapy is safe and have few adverse events.^[11,20,21]^ Fire needle could increase level of adenosine and AMP and ADP to release pain.^[22]^ The internal mechanism to treat the PHN patients is to possibly change the protomics indexes like MFG-ES, Lymphotoxin beta/TNFSF3, IL-19, Neuritin, NCAM-1/CD56, and PECAM-1/CD31 by the Zhuang medicine medicated thread moxibustion.^[16]^

However, there is no available systematic review for the effectiveness and safety of thermotherapy in patients with HZ or PHN. We hope this systematic review can help comprehensively to evaluate its efficacy and safety, and contribute to the management and mechanism of HZ or PHN.

## Author contributions

Guohua Lin is the guarantor of the article and will be the arbitrator when meeting disagreements. All research members participated in developing the criteria and drafting the protocol for this systematic review. YS, JZ, ZkL, and ZL established the search strategy. YS, ZL will independently accomplish the study selection, data extraction, and assess the risk of bias. ZkL, JZ will perform the data syntheses. The subsequent and final versions of the protocol are critically reviewed, modified, and authorized by all authors.

**Conceptualization:** Guohua Lin.

**Data curation:** Zhenke Luo, Jingchun Zeng.

**Formal analysis:** Zhuang Li, Yalin She.

**Funding acquisition:** Guohua Lin, Jingchun Zeng.

**Methodology:** Guohua Lin, Jingchun Zeng.

**Project administration:** Guohua Lin.

**Software:** Yalin She, Zhenke Luo.

**Supervision:** Wenya PEI, Jingchun Zeng.

**Validation:** Zijun Liu.

**Visualization:** Wenya PEI, Zhuang Li.

**Writing – original draft:** Zhuang Li, Yalin She, Zhenke Luo, Zijun Liu.

**Writing – review & editing:** Guohua Lin, Jingchun Zeng.
